# Ultrasound Guided Needle Aspiration versus Surgical Drainage in the management of breast abscesses: a Ugandan experience

**DOI:** 10.1186/1756-0500-5-12

**Published:** 2012-01-06

**Authors:** Alphonce B Chandika, Anthony M Gakwaya, Elsie Kiguli-Malwadde, Phillipo L Chalya

**Affiliations:** 1 Department of Surgery, Weil Bugando University College of Health Sciences, Mwanza, Tanzania; 2 Department of Surgery, Makerere University Kampala, Kampala, Uganda; 3 Department of Radiology, Makerere University Kampala, Kampala, Uganda

**Keywords:** Breast abscess, Ultrasound guided needle aspiration, Surgical drainage, Uganda

## Abstract

**Background:**

Despite breast abscess becoming less common in developed countries, it has remained one of the leading causes of morbidity in women in developing countries. A randomized controlled trial was conducted at Mulago hospital complex in Kampala Uganda to establish whether ultrasound guided needle aspiration is a feasible alternative treatment option for breast abscesses.

**Results:**

A total of 65 females with breast abscess were analyzed, of these 33 patients were randomized into the ultrasound guided needle aspiration and 32 patients in the Incision and drainage arm. The mean age was 23.12, most of them were lactating (66.2%), primipararous (44.6%) with peripheral abscesses (73.8%) located in the upper lateral quadrant (56%).The mean breast size was 3.49 cm. The two groups were comparably in demographic characteristic and breast abscess size. Survival analysis showed no difference in breast abscess healing rate between the two groups (Log rank 0.24 df 1 and *P *= 0.63). Incision and drainage was found to be more costly than ultrasound guided aspiration (cost effective ratio of 2.85).

**Conclusion:**

Ultrasound guided needle aspiration is therefore a feasible and cost effective treatment option for both lactating and non lactating breast abscesses with a diameter up to 5 cm by ultrasound in an immune competent patient

## Background

Breast abscess is a common cause of morbidity in women. While they are less common in developed countries as a result of improved maternal hygiene, nutrition, standard of living and early administration of antibiotics, breast abscess remain a problem among women in developing countries [1]. The treatment of breast abscesses poses a difficult clinical problem [2]. Traditionally, management of breast abscess involves incision and drainage; however this is associated with need for general anesthesia, prolonged healing time, regular dressing, difficulty in breast feeding, and possible unsatisfactory cosmetic outcome [3]. Even with the aggressive approach of incision and drainage combined with use of antibiotics, breast abscess recurrence rate is reported to be between 10 and 38% [2]. Breast abscesses can be treated by repeated needle aspiration with or without ultrasound guidance [4-6]. Ultrasound has been shown to be useful in diagnosis of breast abscesses, guiding needle placement during aspiration and also enables visualization of multiple abscess loculation and thus useful in needle aspiration of breast abscesses [7]. This procedure has been used successful and is associated with less recurrence, excellent cosmetic result and has less costs [8].

Incision and drainage is still the most common mode of treatment for breast abscesses in Uganda. There is no data to compare the outcome of breast abscess treatment when using ultrasound guided needle aspiration versus surgical incision and drainage. The aim of this study was to establish whether ultrasound guided needle aspiration is a feasible alternative treatment option for breast abscesses in Mulago hospital.

## Methods

### Study design and setting

This was a randomized controlled clinical trial with no blinding done between October 2006 and March 2007. The study was a hospital based which was conducted in Mulago hospital complex which is in Kampala city with a population of about 1.2 M people. Mulago is a National referral and teaching hospital in Uganda, it has bed capacity 1500.The study was conducted in the Accident and Emergency (A & E) department and breast outpatient clinic.

### Study subjects

The study included all female patients aged 14 and above who presented to A&E department and Breast Clinic with breast abscess with a diameter of up to a maximum of 5 cm by ultrasound. Patients with recurrent or chronic breast abscess and those with necrotic skin overlying the abscess or abscess already draining were excluded from the study. Patients with clinical features of immune suppression (WHO clinical stage III and IV) and those known to be allergic to penicillin antibiotics were also excluded.

Recruitment of patients was carried out in the Accident and Emergency department, and Breast Outpatient Clinic. Patients who met the inclusion criteria were enrolled into the study. Clinical diagnosis was made basing on the presence of breast pain, swelling, ± fever and presence of a fluctuant tender breast swelling. The patients diagnosed clinically were subjected to ultrasound scan (high frequency linear transducer of 7.5 MHZ) in the radiology department. The diagnosis was confirmed sonographically by the presence of a thick walled echo complex mass, predominantly cystic with internal echoes and septations. The size of the abscess was estimated.

In this study, healing was defined as achieving breast abscess resolution. Breast abscesses resolution was defined as clinically no breast tenderness, swelling or wound at the previous site of the abscess and sonographically complete absence of fluid collection, normal breast glandular and fibro fat tissue with no edema

### Randomization

Patients were randomized to either incision and drainage or needle aspiration arm using computer-generated numbers. A computer program (random generator number, Microsoft excel version 5:0) was used to generate random number list. Patients were assigned to either needle aspiration (A) or incision and drainage (B).The principal investigator randomized patients to either A or B as they presented at the Accident and Emergency department. There was no blinding.

## Treatment procedure and follow up

### Incision and drainage

Patients in the incision and drainage arm were admitted in the Emergency ward and prepared for surgery under general anesthesia in casualty theatre by the principal investigator. In the operation theatre with the patient positioned supine, the breast was swabbed using Chlorhexidine- Cetrimide (Cetrimide 15% w/v, Chlorhexine 1.5%w/v Isopropylalcohol4%w/v) 35 mls in 1 L of water. A skin depth incision was made at the area of maximum fluctuation along skin lines and a sinus forceps used to reach the abscess cavity. Initial pus was swabbed with a sterile pus swab which was transported for Culture and sensitivity. The pus was then evacuated and loculi broken down digitally, the wound was packed with sterile gauze. After recovery, the patient was taken back to emergency ward.

Post operatively the patient was put on analgesics and antibiotics, Diclofenac 75 mg i/m stat, then 50 mg orally for 3 days and Cloxacillin 500 mg 8hry for 10 days respectively. The patient was discharged home the next morning to undergo daily wound dressing at a nearby clinic until the wound heals. Patients whose culture and sensitivity results showed resistance to Cloxacillin were excluded from the study and the antibiotic treatment changed accordingly.

### Ultrasound guided needle aspiration

Patients under the needle aspiration arm were managed in the department of Radiology Ultrasound room as outpatient cases. Under aseptic condition, a small area of skin adjacent to the abscess was anaesthetized by 1% Lignocaine through a 23 G needle. Aspiration was done under ultrasound guidance using a 16 G needle and a 20 mls syringe. Initial aspirated pus was sent for culture and sensitivity. Aspiration was done until there was no significant residual pus. After the procedure the patient was discharged on antibiotics and analgesics, Cloxacillin 500 mg orally 8hry for 10 days and Diclofenac 75 mg i/m stat then 50 mg orally 8hry for 3 days respectively. Similarly patients whose culture and sensitivity results showed resistance to Cloxacillin were excluded from the study and the antibiotic treatment changed accordingly.

In order to minimize non- compliance to treatment in both arms, drugs were provided by the principal investigator to the patients who could not afford buying the drugs. Patients were required to come back with the packs of drugs during follow up visits to countercheck whether the patients had taken the drugs. In both arms, lactating patients were advised to resume breast-feeding on both breasts as soon as possible as they could tolerate the pain as the baby breast feed. The patient's follow up was done at the OPD by the principal investigator on day 7, day 14 and 30 days. At every follow up, clinical assessment of symptoms and signs was done to assess resolution of the abscess.

Ultrasound scan was done to assess radiological resolution of the abscess which was defined as complete absence of fluid collection, normal breast glandular and fibro fatty tissues without edema. In situation where the abscess persisted in case of ultrasound guided needle aspiration, re-aspiration was to be done on day 7, if it still persisted on day 14 it was considered treatment failure and hence converted to the traditional incision and drainage.

Breast abscess recurrence and acceptance were assessed at the last visit (day 30). Patients who had not achieved complete resolution of the breast abscess at the end of the study period were referred to the Breast outpatient clinic for further follow up.

### Cost estimation

All costs were done in Ugandan shillings. Costs incurred by the patients and they included; cost for antibiotics, analgesics, syringes (20 cc) and cannulas (FG 16) used during U.S.S guided aspiration. These were estimated basing on open market price obtained in the local pharmacies. Costs for lodging, professional fee, surgery, anesthesia, amenities, sundries, doctor in care fee and health care fee were estimated basing on the Mulago hospital private patients' charges for the year 2007. Cost for ultrasound guided aspiration was valued basing on the charges as per radiology department for interventional ultrasound guided procedures. Costs for daily dressing for patients in the incision and drainage group was obtained from patient basing on how much she was charged every time she would go for wound dressing at the nearby clinic.

### Data collection and statistical analysis

Data was collected using a structured and coded interviewer administered questionnaire. Administered in the questionnaire were; Age, Parity, Social economic status, Smoking, Time of presentation from onset of symptoms and Size of breast abscess. Outcome variables included; Time to breast abscess resolution, Breast abscess recurrence, Acceptance of ultrasound guided needle aspiration procedure and Cost of the procedures. Statistical analysis was done using SPSS computer software version 11.5. Categorical data was summarized into proportions, percentages and rates. Continuous data was summarized into mean, median, mode, range and standard deviation. Tables were used to present data. Chi-square was used to compare the differences between the two groups where the outcome was categorical and if continuous, *t*-test was used. Statistical significance was defined as a P value of less than 0.05. Survival Analysis using Kaplan-Meier and Cox Regression was used to compare the healing rates between the two groups. For the cost data, costs in each intervention arm were summed up to give the total expenditure per intervention. The cost effectiveness ratio was determined by dividing the total cost of each intervention group by the number of patients successfully treated.

### Ethical issues

Approval to carry out research was obtained from; Faculty of Medicine Research Committee, National Science and Research Council, Mulago Hospital Complex and the department of surgery, Mulago hospital before the commencement of the study.

## Results

**Figure 1:**
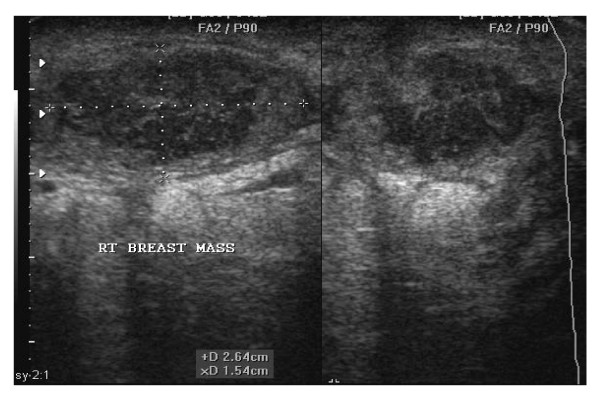
**A sonogram of a 22 yrs old female showing a right breast abscess**. Note the oval shape of the abscess measuring 2.64 cm by 1.54 cm before aspiration under U.S.S guidance.

**Figure 2:**
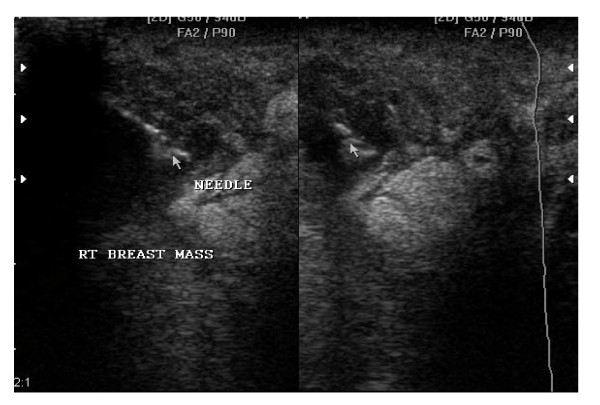
**U.S.S guided needle aspiration of the Right breast abscess of the above patient**. Note the needle in the breast abscess cavity during the aspiration process (see the arrows). Incision and drainage group had a recurrence rate of 3.1% (1/32) during the follow up period. 5 (7.7%) women of the 65 were lost to follow up, 4 patients were from the Ultrasound guided aspiration group and 1 patient from Incision and drainage group. Of the 4 patients in the aspiration group, 2 missed the 2nd visit and the other 2 patients missed the 3rd visit. The patient in the Incision and drainage group lost to follow up in the 3rd visit.

A total of 71 patients with breast abscesses were seen during the study period, of which 65 patients met the inclusion criteria and consented for the study. Six patients were excluded due to their abscesses being already draining pus and others having clinical features of immune suppression. Of the 65 patients, 33 patients were randomized into Ultrasound guided needle aspiration group and 32 patients into incision and drainage group. During the follow up period, Ultrasound guided aspiration had a success rate of 93.1% (27/33) single aspiration, with 6.9% (2/33) re aspirated on the 1st visit due to persistence of the abscess. There was no abscess converted to I & D in the U.S guided needle aspiration group (Figures 1 &2).

**Figure 3:**
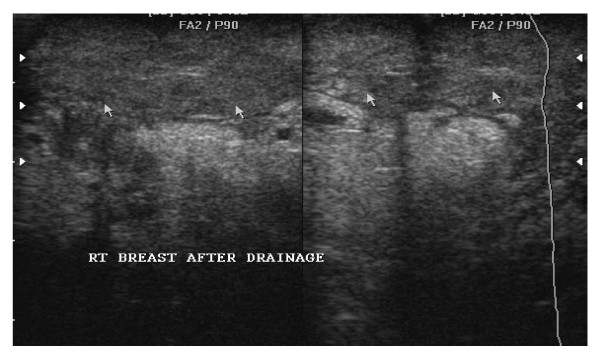
**Right breast after U.S.S guided needle Aspiration of the Abscess of the above patient**. Note the disappearance of the abscess cavity leaving behind inflamed tissues as indicated by arrows.

During the study period, no breast abscess recurrence was observed in the U.S aspiration group, and all the patient (100%) treated by Ultrasound aspiration highly accepted the procedure (Figure 3).

In both groups majority of patients were healed by the 3rd visit that is 65.5% (19) for Ultrasound guided aspiration group and 58.1% (18) for the Incision and drainage group. There was no difference in healing rate between the two study arms at all the three visits (Table 1).

**Table 1:** Healing rates per group

Study group	A	B	Odds ratio	95%CI	P-value
**Visit 1(**Day 7**)**	3% (1)	9.4% (3)	0.30	0.03-3.07	0.29

**Visit 2 (**Day 14**)**	29% (9)	21.9% (7)	1.46	0.47-4.58	0.51

**Visit 3(**Day 30**)**	65.5% (19)	58.1% (18)	1.37	0.48-3.91	0.55

Demographic characteristics, clinical presentation of patients, size, shape and location of the abscess had no effect on the healing rate between the two study groups (*P *> 0.001) (Table 2).

**Table 2:** Mean Healing time of the two study arms at different variables

Variable	Mean healing time in weeks		Mean Difference	95%CI	P-Value
				
	US Aspiration	I&D			
**Age in yrs**					
≤ 20	27.5	30.0	-2.46	-6.2-1.3	0.19
> 20	21.5	19.9	1.61	-4.8-8.1	0.61

**Symptoms**					
≤ 7 days	20.6	22.0	-1.42	-11.8-8.9	0.77
> 7 days	28.7	24.0	4.70	-0.7-10.2	0.09

**B/feeding**					
Yes	26.1	25.8	0.25	-4.9-5.3	0.92
No	22.5	15.8	6.70	-2.5-15.9	0.15

**Parity**					
Primiparous	26.0	26.4	-0.45	-7.1-6.2	0.88
Multiparous	21.3	21.6	-0.35	-8.1-7.4	0.93
Nulliparous	24.7	30.0	-5.33	-28.3-17.6	0.58

**Const.symp2**					
Yes	26.8	26.0	0.80	-8.4-9.9	0.83
No	23.7	22.9	0.80	-4.6-6.2	0.62

**B/affected 3**					
Right	23.3	24.1	-0.73	-7.8-6.3	0.83
Left	24.9	23.3	1.55	-4.9-7.9	0.62

**Q/affected 4**					
LUQ 5	23.6	20.3	3.26	-3.3-9.9	0.32
MUQ 6	18.0	27.3	-9.33	-19.9-1.3	0.08
LLQ 7	24.7	30.0	-5.33	-51.2-40	0.67
MLQ 8	25.7	31.0	-4.0	-49.2-39	0.62

**SizeB/Absc 9**					
≤ 3 cm	15.3	13.5	1.74	-5.0-8.5	0.59
> 3 cm	27.1	30.0	-2.90	-5.93-0.11	0.06

**Shape**					
Oval	22.0	30.0	-8.0	-18-3.0	0.11
Irregular	24.8	23.1	-3.7	-3.7-71	0.52
Multiloculated	24.6	14.0	10.7	-35.2-56	0.42

**Location**					
Subareolar	17.5	25.4	-7.8	-20.2-4.5	0.18
Periphery	26.8	23.1	3.7	-1.2-8.6	0.14
Indeterminate	22.0	30.0	-8.0	-184-168	0.67

### Survival analysis

**Figure 4:**
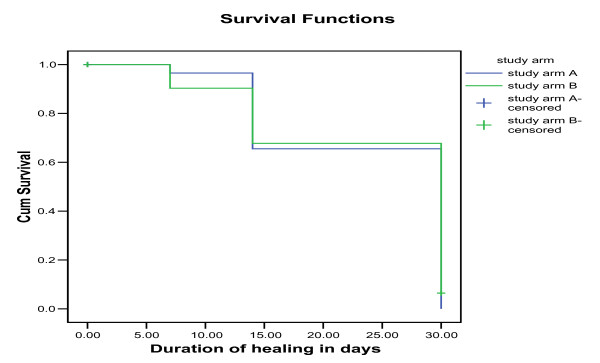
**Survival Function**.

**Figure 5:**
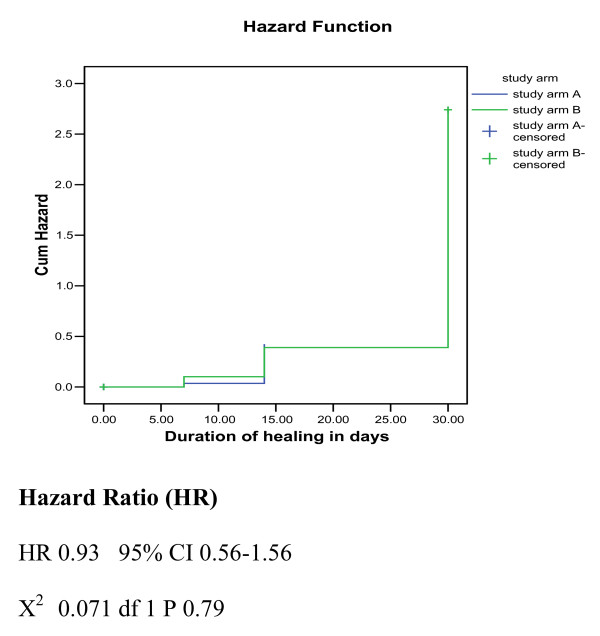
**Hazard Function**.

The duration of healing for study group A was 24.24 days and for group B was 24.16 days (Table 3). The probability of not healing was equal in the first week in both study arms. In the second week, the probability of not healing was slightly higher in group A than B. While in the third week, the probability of not healing was slightly higher in group B than A. Statistically there is no difference in the probabilities of not healing between patients in group A and B (Log rank: 0.24, df 1 P 0.63) (Figure 4). The Hazard rate of patients in Arm A was 0.93 times less the hazard rate of group B and this was not statistically significant (*P *> 0.001) (Figure 5).

**Table 3:** Descriptive analysis

Study Arm	Mean duration of survival (95% CI)	Median duration of survival (95% CI)
A	24.24 (21.27-27.22)	30.00

B	24.16 (21.06-27.27)	30.00 (27.74-32.26)

### Cost-effectiveness analysis

The number of patients effectively treated by Ultrasound guided Needle aspiration (excluding loss to follow up) were 29 and for incision and drainage were 31 patients (Table 4). The cost effectiveness ratio for incision and drainage arm was 2.85 times that of Ultrasound guided needle aspiration.

**Table 4:** Cost-Effectiveness analysis

Category	Intervention	
	**U.S. Guided Needle aspiration**	**Incision & Drainage**

Admission		620000

Professional fee	2900000	3100000

Preoperative fee		620000

Surgery fee		6100000

Anesthetic fee		3200000

Amenities	580000	620000

Sundries	290000	310000

Lodging		930000

Intervention U.S.S	870000	

Cannula (FG 16)	43500	

Syringe 20cc	29000	

Drugs	246500	259600

Dr. in charge care fee	290000	310000

Health care fee	290000	310000

Wound dressing cost		489500

Total	5539000	16869100

Number of patients	29	31

Cost-effectiveness ratio	191000	544164.5

## Discussion

The breast is one of the sex organs of a female, in case of breast disease care should be taken to insure that its beauty is minimally compromised in order to preserve its value and function. Despite of breast abscess becoming less in developed countries due to improved maternal hygiene, nutrition, standard of living and early use of antibiotics, breast abscess remain a problem among women in developing countries [1].

Treatment of breast abscess traditionally has been incision and drainage however this has been found to be associated with possible unsatisfactory cosmetic outcome, difficult in breast feeding and needs general anesthesia, prolonged healing time, and regular dressing [3]. Repeated aspiration with or without ultrasound guidance has been found to be another treatment option for breast abscess and this has been reported to be associated with less recurrence, excellent cosmetic result and has less costs [4-6,8].

This study was conducted to establish whether ultrasound guided needle aspiration is a feasible alternative treatment option for the breast abscess in Mulago hospital.

This discussion is based on 65 patients with breast abscesses randomized into 33 for Ultrasound guided aspiration and 32 for incision and drainage intervention. The healing rate, recurrence, cost effectiveness were compared between the two groups and acceptance of Ultrasound guided needle aspiration was assessed.

Healing rate of the two groups had no statistically significant difference both overall and at each visit (Log rank: 0.24 df 1 P-0.63), this was similar with what was found elsewhere [4].This similarity in the healing rate between the two treatment option could be explained by the fact that regardless of the way pus is removed from the cavity (that is incision and drainage, needle aspiration or spontaneous rupture onto the skin surface) the healing process is the same which is by collapse of the cavity wall and adherence to one another by fibrin, later by granulation tissue. The remaining bacteria destroyed by polymorphs [9].

There was no recurrence of breast abscesses observed in the Ultrasound guided needle aspiration group during the study period. There was 3.1% (1/32) recurrence rate observed in the incision and incision group. However this recurrence rate was far less than 31% recurrence in the incision and drainage group which has been reported in another study [8].This small recurrence rate observed may have been resulted from a short follow up period and it was not possible to compare the recurrence rate of the two study groups.

All the patients treated with Ultrasound guided needle aspiration highly accepted this modality (100%).This was consistence with what other studied found [8,10-12].This high acceptance rate may have been resulted from the convenience of the procedure which was an outpatient one, having no wound to nurse and absence of scar after healing.

The cost effectiveness ratio of Ultrasound guided aspiration was found to be much less than that of Incision and Drainage, thus indicating that Ultrasound guided aspiration provides savings to the hospital and the patient, hence more cost effective than Incision and Drainage. This was consistence with what was found elsewhere [11,13]. Since Ultrasound guided aspiration is an outpatient procedure as opposed to the Incision and Drainage which is inpatient procedure. Studies done to compare outpatient versus inpatient surgical procedures showed that outpatient procedures were cost effective [14,15].

## Conclusion

There is no difference in terms of healing rate of breast abscess between Ultrasound guided aspiration and surgical incision and drainage, Ultrasound guided needle aspiration is highly accepted by women with breast abscesses in Mulago hospital. Ultrasound guided aspiration is more cost effective than Incision and Drainage in management of breast abscess, therefore Ultrasound guided needle aspiration is an effective treatment option for both lactating and non lactating breast abscess.

## Competing interests

The authors declare that they have no competing interests.

## Authors' contributions

ABC conceived and designed the study and did the literature search, coordinated the write-up, editing and submission of the article. AMG & KEM coordinated the write-up, editing and supervised the study. PLC participated in the literature search, writing of the manuscript and editing. All the authors read and approved the final manuscript.
